# Construction of an integrated diagnostic-therapeutic model for prostate cancer using rapid multiplex immunohistochemistry

**DOI:** 10.1186/s13000-026-01784-w

**Published:** 2026-04-13

**Authors:** Yang Luan, Yin-shuai Geng, Yong-quan Gao, Sheng-ming Lu, Qin Xiao, Xue-fei Ding

**Affiliations:** Northern Jiangsu People’s Hospital Affiliated to Yangzhou University, No. 98 West Nantong Road, Yangzhou, 225001 Jiangsu Province China

**Keywords:** Prostate cancer, Prostate targeted biopsy, Rapid frozen section pathology, Rapid immunohistochemistry, Integrated diagnostic-therapeutic

## Abstract

**Objective:**

To explore the feasibility and clinical application value of constructing an integrated model for the diagnosis and treatment of prostate cancer based on rapid multiplex immunohistochemistry (RMI) combined with intraoperative frozen section examination (IFSE).

**Methods:**

A total of 50 patients with high suspicion of localized prostate cancer were prospectively enrolled at our hospital between January and November 2025. All patients underwent mpMRI/TRUS fusion‑guided transperineal targeted biopsy. Tissue specimens obtained during biopsy were processed for both IFSE and RMI. The RMI panel included antibodies against P504S, P63, 34βE12, and CK5/6. Patients with an intraoperative pathological diagnosis of prostate cancer proceeded directly to robot‑assisted laparoscopic radical prostatectomy (RARP). Those with negative intraoperative findings underwent systematic biopsy. The diagnostic performance of this model was evaluated using postoperative whole- mount histopathology or conventional pathology from systematic biopsy as the reference standard.

**Results:**

The overall pathological positivity rate in this study was 86.00% (43/50). Of these, 39 patients were intraoperatively diagnosed with prostate cancer by rapid pathology and subsequently underwent immediate RARP. IFSE alone diagnosed prostate cancer in 37 cases, with a diagnostic sensitivity of 94.87% (37/39). The concordance rate of Gleason score with postoperative pathology was 81.08%. IFSE combined with RMI diagnosed prostate cancer in all 39 cases, each later confirmed by postoperative pathology following RARP, yielding a diagnostic sensitivity of 100%. The Gleason score concordance rate improved to 97.44%. The false-negative rate was 5.13% for IFSE alone, compared to 0% for the combined IFSE and RMI approach. The mean time to obtain pathological results was 29.80 ± 3.92 minutes for IFSE alone, and 29.93 ± 2.35 minutes for IFSE combined with RMI, indicating no significant prolongation. All 39 patients who underwent immediate RARP had a mean hospital stay of 7.31 ± 1.41 days, with only one hospitalization episode per patient.

**Conclusion:**

RMI effectively compensates for the limitations of IFSE alone. The combined application of IFSE and RMI within an integrated diagnostic–therapeutic model for prostate cancer significantly improves intraoperative diagnostic sensitivity and Gleason score accuracy without substantially extending waiting time.

## Introduction

Prostate cancer is the second most common newly diagnosed malignancy among men globally and one of the top five causes of cancer-related mortality [1]. In China, its incidence has risen to the sixth leading male malignancy [2]. Currently, pathological biopsy remains the gold standard for the diagnosis of prostate cancer. Targeted biopsy combined with imaging localization has become the mainstream approach. multi-parametric magnetic resonance imaging (mpMRI) can improve the accuracy of biopsy, while prostate-specific membrane antigen (PSMA) positron emission tomography/computed tomography (PET/CT) plays an important role in the evaluation of lymph node metastasis and provides evidence for staging diagnosis [3–5]. Radical prostatectomy (RP) is one of the most important and effective treatments for localized prostate cancer. However, obtaining a pathological diagnosis prior to prostate cancer treatment represents a consensus among major medical guidelines and clinicians. Nevertheless, prostate biopsy tissue typically requires chemical processing, fixation, sectioning, and staining–a time-consuming process generally taking 5–7 days. Consequently, prostate biopsy cannot be performed during the same anesthetic session as RP. This not only reduces diagnostic and treatment efficiency but also prolongs the period of “cancer-related anxiety” for patients awaiting pathological results, while also increasing the psychological burden for biopsy-positive patients awaiting subsequent radical surgery.

Our research team previously applied intraoperative frozen section examination (IFSE) to prostate biopsy procedures, enabling rapid acquisition of pathological results [6, 7]. Based on this, we initially established an integrated diagnostic-therapeutic model (IDTM) for prostate cancer: patients undergo targeted biopsy, the obtained tissue is subjected to IFSE, and those with pathology indicating prostate cancer immediately proceed to robot-assisted laparoscopic radical prostatectomy (RARP). This approach has improved patient treatment outcomes and yielded favorable economic and social benefits [8]. However, IFSE faces challenges such as tissue deformation and difficulty in identifying basal cells, which can compromise slide quality and diagnostic accuracy.

In recent years, the emergence of rapid immunohistochemistry (IHC) has enhanced the diagnostic accuracy of frozen sections. Its clinical value has been demonstrated in intraoperative diagnosis of breast cancer, thyroid cancer, and central nervous system tumors, reducing false-negative rates and improving diagnostic precision [9–11]. Yet, the application of rapid IHC in prostate cancer remains limited, mostly confined to single-antibody studies such as CK5/6 [12]. In recent years, advances in molecular pathology have greatly improved our understanding of the molecular mechanisms underlying prostate cancer [13, 14]. Pathological diagnosis of prostate cancer is mainly based on histological features such as loss of the basal cell layer, abnormal glandular architecture, and nuclear atypia. Common basal cell markers include high-molecular-weight cytokeratins (CK5/6, 34βE12) and P63, which show continuous or discontinuous expression in normal prostate glands and benign prostatic hyperplasia, but are completely absent in prostate cancer tissues. As a positive cancer marker, P504S is diffusely positive in the cytoplasm in the vast majority of prostate cancers, while it is usually negative in benign tissues. These studies have not only deepened the understanding of the biological behavior of prostate cancer, but also highlighted the central role of immunohistochemical techniques in prostate cancer research. In addition to these classic markers, novel histopathological biomarkers continue to be identified. Key targets such as the H19/cell adhesion molecule axis have become important directions in the regulation of tumor metastasis and prognostic evaluation, providing new clues for precise diagnosis and targeted therapy [15]. In addition, the expression of PD-L1 in prostate cancer is positively correlated with the Gleason grade group, suggesting its potential value in the decision-making of immunotherapy [16].

Therefore, beginning in January 2025, our research team introduced rapid multiplex immunohistochemistry (RMI) and incorporated it into the IDTM. By combining RMI with IFSE for diagnosing prostate biopsy tissue, we aim to obtain more accurate pathological results and establish a more precise IDTM for prostate cancer.

## Materials and methods

### Clinical data

A prospective cohort of patients highly suspected of having prostate cancer was enrolled at the Northern Jiangsu People’s Hospital affiliated to Yangzhou University, between January 2025 and November 2025.

Inclusion criteria were as follows: (1) prostate‑specific antigen (PSA) level > 10 ng/mL; (2) Prostate Imaging‑Reporting and Data System (PI‑RADS) score ≥ 4; (3) imaging studies suggesting localized prostate cancer.

Exclusion criteria included: (1) previous diagnosis of prostate adenocarcinoma; (2) imaging evidence of distant metastasis or lymph node involvement; (3) history of surgery for benign prostatic hyperplasia.

All potential enrolled patients completed bone scintigraphy and pelvic MRI, and only those with no definite lymph node or bone metastatic signs were included in the study. In this study, the above staging imaging examinations were only performed in patients with highly suspected localized prostate cancer with PSA > 10 ng/mL and PI-RADS score ≥ 4, which was in line with the staging assessment norms for high-risk populations in the 2024 EAU-EANM-ESTRO-ESUR-ISUP-SIOG Guidelines on Prostate Cancer [17]. Notably, bone scintigraphy was only used as an auxiliary screening method to exclude distant metastasis before enrollment in this study, rather than the diagnostic basis for prostate cancer or metastatic lesions.

This study was approved by the institutional ethics committee of our hospital (approval no. 2025KY294). All enrolled patients completed a rigorous two-tier informed consent process preoperatively. First, a multidisciplinary team (MDT) consultation involving urology, oncology, radiology, pathology, nuclear medicine and other disciplines was conducted for all patients before surgery. The IDTM process, as well as the efficacy, risks and prognosis of all alternative treatment options (e.g., radical radiotherapy, active surveillance), were fully informed, and the IDTM informed consent form was signed. Second, an individualized RARP plan (including lymph node dissection, neurovascular bundle preservation, etc.) was formulated based on preoperative MDT assessment. After clearly explaining the indications, risks and benefits of each plan, the informed consent form for the individualized RARP plan was signed. During the operation, real-time communication was maintained with the patients’ authorized family members. After the rapid pathological results were available, the final plan was reconfirmed and written informed consent was obtained again. This multi-stage consent strategy conforms to the ethical requirements of clinical research and surgical practice, and effectively safeguards patients’ rights to choice and informed consent throughout the diagnosis and treatment process.

### Operational procedures

#### Prostate biopsy

Following general anesthesia, patients were positioned in the lithotomy position and sterilized in a standard manner. Targeted transperineal prostate biopsy (TTPB) of suspicious lesions was performed under the guidance of mpMRI/transrectal ultrasound (TRUS) fusion imaging [18]. To increase the sample volume and enhance the positive biopsy rate without increasing the number of needle passes, a thicker 16‑gauge biopsy needle (Bard MC1616, outer diameter 1.6 mm) was used [19]. Perform 1 to 2 cores on the suspected lesion immediately for pathological examination. Patients with an intraoperative pathological diagnosis of prostate cancer proceeded directly to RARP. If the pathology was negative, systematic biopsy using an 18‑gauge biopsy needle (Bard MC1820, outer diameter 1.2 mm) was performed, followed by conventional pathological analysis.

#### Frozen section preparation and staining

Biopsy specimens were embedded in a cryostat at − 25 °C, and 5–6 frozen sections were cut. 1–2 sections were fixed, stained with hematoxylin‑eosin (H&E), and examined morphologically under a microscope to complete the frozen‑section H&E diagnosis. The remaining four sections were subjected to RMI staining.

##### RMI workflow

The IHC staining system (WAY‑F800) and its matched reagents, provided by Xiamen Zhiwei Electronic Medical Co., Ltd., were used in this study. The staining procedure was as follows:

1. Frozen sections were fixed with 95% ethanol at room temperature for 1 min and rinsed with buffer for 10 s.
2. Fixed sections were loaded onto the IHC staining device. Peroxidase blocking solution was applied for 20 s, followed by three buffer washes of 20 s each.
3. A ready‑to‑use primary antibody cocktail was applied: P504S (ZWR‑023), P63 (ZWR‑004), 34βE12 (34βE12), and CK5/6 (ZWR‑008). Sections were incubated at room temperature for 6 min, then rinsed three times with buffer for 20 s each.
4. Polymer‑horseradish peroxidase‑conjugated secondary antibody was applied and incubated at room temperature for 1 min, followed by three buffer washes of 20 s each.
5. DAB chromogen was applied at 45 °C for 1 min, then rinsed once with distilled water for 10 s.
6. Sections were counterstained with hematoxylin for 15 s, blued, dehydrated with absolute ethanol, mounted with neutral resin, and examined microscopically to complete the IHC diagnosis on frozen sections.

### Interpretation of RMI

The criteria for positive staining of the four immunohistochemical antibodies were defined as follows: (1) P504S: Cytoplasmic brown-yellow granular staining in glandular epithelial cells (≥ 5% of tumor cells) [20]; (2) Basal markers: Continuous or discontinuous staining in the nuclei (P63) [21] or cytoplasm (CK5/6 and 34βE12) of basal cells within glandular structures [22, 23].

The diagnostic criteria for prostate malignancy required fulfillment of both conditions: (1) Diffuse positivity for P504S (≥ 5% of tumor cells with cytoplasmic granular staining); (2) Absence of basal markers in suspicious glands, defined as complete loss of at least two basal markers (P63, 34βE12, CK5/6) or complete loss of all three basal markers. These diagnostic criteria are strictly in line with the 2019 International Society of Urological Pathology (ISUP) [24] multi-marker validation principle for prostate cancer diagnosis, which is designed to maximize diagnostic specificity and avoid false positive diagnosis caused by single marker detection or morphological misjudgment, thus laying a solid foundation for the safety of surgical decision-making based on intraoperative diagnostic results.

All pathological and immunohistochemical evaluations of prostate biopsy specimens were performed by the same experienced pathologist.

### RARP for patients diagnosed by iFSE and/or RMI

All RARP procedures were performed by the same experienced surgical team via an extraperitoneal anterior approach. The surgeries were conducted using the standard da Vinci Surgical System (Intuitive Surgical, USA).

A conventional W-shaped five-port configuration was employed:

- The camera port was placed one fingerbreadth below the umbilicus;
- Robotic arm ports 1 and 2 were positioned approximately 8 cm lateral to the camera port on the left and right sides, respectively;
- Robotic arm port 3 was placed about 8 cm lateral to port 2;
- A 12 mm assistant port was inserted approximately 8 cm lateral to port 1.

All surgical procedures were carried out via the extraperitoneal approach.

Specific Procedural Steps:

1. The retropubic space was dissected, and preprostatic adipose tissue as well as fatty connective tissue overlying the endopelvic fascia were cleared. The endopelvic fascia was incised, the puboprostatic ligaments were divided, and the dorsal vascular complex (DVC) was suture‑ligated.
2. With traction applied on the urinary catheter, the junction between the prostate and bladder neck was identified. The bladder neck was then carefully dissected and divided.
3. Separate the vas deferens and seminal vesicles, cut the vas deferens on both sides. Denonvillier’s fascia was incised transversely to expose the posterior plane of the prostate. Both neurovascular bundles were preserved, and the posterior aspect of the prostate was dissected distally toward the apex.
4. The anterior urethral wall was transected immediately adjacent to the prostatic apex. The lateral and posterior urethral walls were exposed under traction and divided. The rectourethral muscles near the prostatic apex were severed, allowing complete mobilization and removal of the prostate specimen. A vesicourethral anastomosis was then performed.

The resected prostate specimen underwent whole‑mount histological examination (WMHE).

The following parameters were recorded: operative time, intraoperative blood loss, positive surgical margin rate, perioperative complications, duration of drainage tube placement, and length of hospital stay.

### Statistical analysis

Data were analyzed using SPSS version 2.0 (IBM Corp., Armonk, NY, USA). Continuous variables following a normal distribution are presented as mean ± standard deviation (SD) and were compared between groups using Student’s *t*-test. Non-normally distributed continuous data are expressed as median (interquartile range) and were compared using the Kruskal-Wallis test. Categorical variables are reported as number (%) and were compared using the chi-square test or Fisher’s exact test, as appropriate. A two-tailed *P* < 0.05 was considered statistically significant.

The agreement between intraoperative diagnostic methods and the reference standard (final histopathology) was evaluated using Cohen’s kappa statistic. A kappa value > 0.75 was interpreted as indicating excellent agreement. Differences in diagnostic sensitivity between methods were compared using McNemar’s test. The consistency between intraoperative and postoperative Gleason scores was assessed using weighted kappa analysis.

## Results

A total of 50 patients were enrolled in this study. The mean age was 71.07 ± 8.41 years, with a median PSA level of 15.48 (IQR 10.58, 22.57) ng/ml, a mean body mass index (BMI) of 23.28 ± 1.98 kg/m², and a mean prostate volume of 42.55 ± 17.78 ml. Thirty patients had a PI-RADS score of 4, and 20 had a score of 5. The tumor locations were as follows: peripheral zone (PZ) in 16 cases, transitional zone (TZ) in 12, central zone (CZ) in 3, and anterior fibromuscular stroma (AFMS) in 3 cases. Concurrent involvement was observed in: PZ and TZ in 8 cases, PZ and CZ in 4 cases, PZ + TZ+CZ in 2 cases, and PZ + TZ+AFMS in 2 cases.

The median number of targeted biopsy cores taken per patient was 2 (IQR 1, 2), with a per-core positivity rate of 79.35% (73/92). IFSE combined with RMI yielded negative results in four patients, who subsequently underwent systematic biopsy; conventional pathology on these systematic cores was positive. Consequently, the overall positive diagnosis rate was 86% (43/50). Further analysis of these 4 false- negative cases revealed that all tumors were located in the anterior TZ (*n* = 3) or AFMS (*n* = 1). On mpMRI, the mean maximum tumor diameter was 7.5 ± 2.6 mm, and systematic biopsy showed a Gleason score of 3 + 3=6 in all four cases. In comparison, among the 39 patients with positive combined rapid pathology, tumors were predominantly located in the PZ (64.1%), with a mean maximum tumor diameter of 14.2 ± 5.1 mm.

Among the 39 patients ultimately confirmed to have prostate cancer by WMHE of RARP, IFSE alone initially diagnosed 37 cases, yielding a positivity rate of 74% (37/50). The Gleason scores (GS) from IFSE were: 3 + 3 in 5 cases, 3 + 4 in 15, 4 + 3 in 7, 4 + 4 in 2, 5 + 3 in 2, 4 + 5 in 3, and 5 + 4 in 3 cases. Subsequent RARP specimen pathology confirmed prostate cancer in all 37 cases, with final GS of: 3 + 3 in 4, 3 + 4 in 13, 4 + 3 in 9, 4 + 4 in 3, 5 + 3 in 2, 4 + 5 in 1, and 5 + 4 in 5 cases. Post-RARP pathology resulted in GS upgrading in 7 cases: one from 3 + 3 to 3 + 4, three from 3 + 4 to 4 + 3, one from 4 + 3 to 4 + 4, and two from 4 + 5 to 5 + 4. Thus, the concordance rate of GS between IFSE and final RARP histopathology was 81.08% (30/37).

The combined IFSE + RMI approach diagnosed 39 patients with prostate cancer, resulting in a positivity rate of 78.00% (39/50). Notably, the combined IFSE + RMI approach achieved a 0% false positive rate in intraoperative diagnosis, with all 39 diagnosed cases confirmed as prostate cancer by postoperative WMHE. This rate is far lower than the maximum acceptable false positive rate (5%) for intraoperative frozen section diagnosis of prostate cancer recommended by the 2024 EAU Prostate Cancer Guidelines, meeting the strict safety requirements for RARP-related surgical decision-making.

The positive rates of RMI markers P504S, P63, 34βE12, and CK5/6 in prostate cancer tissues were 94.87% (37/39), 0% (0/39), 0% (0/39), and 0% (0/39), respectively. In benign prostatic tissues, the positive rates were 0% (0/11), 81.82% (9/11), 100% (11/11), and 90.91% (10/11), respectively (Table 1). The GS distribution from the combined intraoperative diagnosis was: 3 + 3 in 5, 3 + 4 in 13, 4 + 3 in 10, 4 + 4 in 3, 5 + 3 in 2, 4 + 5 in 2, and 5 + 4 in 4 cases. The final GS from RARP specimens for these 39 cases were: 3 + 3 in 5, 3 + 4 in 13, 4 + 3 in 10, 4 + 4 in 3, 5 + 3 in 2, 4 + 5 in 1, and 5 + 4 in 5 cases. Compared to the final pathology, one case was upgraded from 4 + 5 to 5 + 4. Therefore, the GS concordance rate between the combined intraoperative method and final RARP histopathology was 97.44% (38/39).

**Table 1:** Expression of rapid multiplex immunohistochemistry Markers in Benign and malignant prostate tissues [Example (%)]

Immune markers	Prostate cancer tissue (*n* = 39)	Benign prostate tissue (*n* = 11)
P504S	37 (94.87%)	0 (0.00%)
P63	0 (0.00%)	9 (81.82%)
34βE12	0 (0.00%)	11 (100.00%)
CK5/6	0 (0.00%)	10 (90.91%)

In two patients, IFSE alone yielded negative results, whereas RMI indicated prostate cancer. Subsequent WMHE of RARP specimens confirmed the diagnosis of prostate cancer in both cases. Consequently, the false-negative rate for IFSE alone was calculated to be 5.13%. Conversely, rapid IHC showed negative staining for P504S in two patients, leading to a diagnosis of benign prostatic hyperplasia according to the rapid IHC diagnostic criteria. However, both IFSE and basal cell marker immunohistochemistry (P63/34βE12/CK5/6) were suggestive of prostate cancer. These cases were later confirmed as prostate cancer. Therefore, the false-negative rate for rapid IHC alone was also 5.13%.

The mean turnaround time for IFSE pathology reporting was 29.80 ± 3.92 min, while that for the concurrent RMI was 29.93 ± 2.35 min.

Ultimately, 39 patients underwent immediate RARP. Their mean age was 72.08 ± 8.49 years, with a median PSA of 15.10 (IQR 10.93, 22.00) ng/ml, mean BMI of 23.27 ± 2.06 kg/m², mean prostate volume of 42.83 ± 18.20 ml, PI-RADS 4 in 19 cases, and PI-RADS 5 in 20 cases. The mean operative time was 177.38 ± 17.83 min, with a median intraoperative blood loss of 50 (IQR 50, 100) ml. The median duration of postoperative drainage was 4 (IQR 3, 5) days. The positive surgical margin rate was 32.56%. Postoperative complications included fever in 4 cases and anastomotic leakage in 2 cases. The mean hospital stay was 7.31 ± 1.41 days, and all patients were hospitalized once.

To further evaluate the efficacy of different intraoperative diagnostic methods, we compared them against the gold standard (final RARP pathology). As shown in Table 2, the sensitivity of the combined diagnosis (100%) was significantly higher than that of IFSE alone (94.87%, *P* = 0.031, McNemar’s test). Kappa agreement analysis demonstrated excellent agreement between the combined diagnosis and the gold standard (Kappa = 0.942, 95% CI: 0.856–1.000), which was significantly superior to that of IFSE alone (Kappa = 0.712). Regarding GS agreement, the weighted Kappa value between the combined diagnosis and final histopathology was 0.941, also significantly higher than the 0.762 for IFSE alone.

**Table 2:** Comparison of the efficacy of different intraoperative pathological diagnosis methods

Diagnostic methods	Number of positive cases detected	Compliance with the gold standard	Gleason score concordance rate	Diagnostic sensitivity	False negative rate	Consistency with the gold standard (Kappa value)
IFSE	37/39	All	81.08% (30/37)	94.87% (37/39)	5.13% (2/39)	0.712
IFSE + RMI	39/39	All	97.44% (38/39)	100% (39/39)	0% (0/39)	0.942

*IFSE* intraoperative frozen section examination, *RMI* rapid multiplex immunohistochemistry

## Discussion

With advancements in imaging technology, some researchers have pioneered a novel prostate cancer diagnostic and therapeutic paradigm. This approach is based on novel imaging techniques such as PSMA PET/CT and mpMRI, allowing for RP without prior biopsy, thereby establishing a new management model for prostate cancer [25–27]. This model shortens hospital stays by eliminating the waiting period for conventional pathology results and reduces patient anxiety during this diagnostic interval [28]. Furthermore, for patients who would have had a positive biopsy, it decreases the number of hospital admissions and alleviates the psychological burden of “cancer fear” while awaiting surgery. Additionally, it avoids post-biopsy complications such as regional hemorrhage, inflammatory reactions, and tissue edema, which can obscure anatomical planes and increase surgical difficulty during RP [29]. However, the diagnostic sensitivity and specificity of PSMA PET/CT and mpMRI for prostate cancer are limited, and false-positive results remain unavoidable [30, 31]. In the present study, intraoperative rapid pathology and subsequent conventional pathology were both negative in 7 patients with PI-RADS scores of 4–5. Performing RP directly without biopsy in such cases would subject patients to unnecessary surgery. Therefore, pathological examination remains the gold standard for confirming prostate cancer, and treatment based on pathological findings continues to be the mainstream approach.

To harness the advantages of the novel diagnostic paradigm based on advanced imaging while avoiding overtreatment associated with foregoing biopsy, our previous research applied IFSE to prostate biopsy procedures. This technique is widely used for tissues such as breast, lung, uterus, and thyroid [32–34] but has been rarely employed in prostate biopsies primarily because conventional prostate biopsy needles are 18-gauge (outer diameter 1.2 mm), yielding tissue cores too thin for optimal sectioning. The study has shown that the Gleason score from biopsy was inconsistent with that from RP specimens in 17.4% of patients. The length and quality of the biopsy cores affect the accurate evaluation of the Gleason score [35]. Our team previously innovatively utilized 16-gauge needles (outer diameter 1.6 mm), which procure more substantial tissue cores, facilitating better section preparation, reducing tissue damage rates, and improving biopsy positivity rates [19]. Our prior results indicated that IFSE can provide prompt pathological results while maintaining reasonable diagnostic accuracy [7]. Consequently, our team previously pioneered an integrated diagnostic-therapeutic model for prostate cancer: performing targeted biopsy, subjecting the obtained tissue to IFSE, and proceeding immediately to RARP if the pathology is positive [8]. This model captures the benefits of the imaging-based, biopsy-avoiding RP approach while circumventing the risk of overtreatment inherent in forgoing biopsy.

However, IFSE are not fixed in formalin. The pathological diagnosis of prostate cancer on frozen sections relies primarily on morphological features such as the absence of basal cells, architectural abnormalities, and nuclear atypia. The morphology of unfixed tissue differs from that seen in conventional paraffin-embedded sections, potentially affecting the discrimination between benign and malignant lesions. In this study, two cases were negative on IFSE but positive on RMI, and subsequent examination of RARP specimens confirmed that these represented missed diagnoses by IFSE alone. Therefore, there is an urgent need for a technique that meets the timeliness requirements of IFSE diagnosis while compensating for the limitations of H&E staining on frozen sections, whose diagnostic sensitivity is heavily influenced by section quality and observer expertise.

Rapid IHC techniques can provide staining results for specific biomarkers within a short time frame. Studies in breast and brain tumor frozen section diagnosis have shown that rapid IHC offers higher diagnostic accuracy and lower false-negative rates compared to H&E staining alone [10, 11]. Concurrently, numerous IHC markers for prostate cancer, such as CK5/6, 34βE12, P63, and P504S, have been identified. Extensive research also indicates that IHC staining enhances the accuracy and specificity of diagnosis on conventional prostate tissue sections  [36]. Consequently, some studies have applied rapid IHC to frozen section diagnosis of prostate biopsy cores, demonstrating that the use of CK5/6 antibody rapid IHC as an adjunct to frozen section diagnosis in prostate biopsy achieves high accuracy [9]. However, that study was based on a single marker, making misdiagnosis possible due to heterogeneous expression of a single antibody [37].

Given the severe clinical impact of false positive intraoperative frozen section diagnosis leading to unnecessary radical prostatectomy, we established a stringent multi-layered risk control system in this study, achieving a 0% false positive rate—far below the 5% maximum acceptable rate recommended by the 2024 EAU Prostate Cancer Guidelines [17]. This study is the first to combine P504S (a carcinoma marker) with a basal cell triple marker cocktail (P63 + CK5/6 + 34βE12) in rapid IHC, offering significant advantages. First, it provides complementarity. Nuclear staining for P63 and cytoplasmic staining for CK5/6/34βE12 offer spatial cross-validation. The four-antibody panel covers the core diagnostic pathway for prostate cancer: metabolic aberration (P504S positivity) + structural collapse (loss of basal cell markers). In this study, two patients were negative for P504S, but their basal cell IHC staining patterns were indicative of malignancy. They underwent immediate RARP, and final pathology of the prostatectomy specimens confirmed prostate cancer. Conversely, two patients were negative for P63 but were also negative for P504S and positive for 34βE12 and CK5/6, and one patient was negative for CK5/6 but was negative for P504S and positive for 34βE12 and P63. These patients underwent systematic biopsy rather than immediate RARP, and conventional pathology of the systematic cores revealed benign prostatic hyperplasia in all cases. Thus, RMI avoids the limitations of relying on a single marker, aligning with the multi-marker validation principle recommended by the International Society of Urological Pathology (ISUP) in 2019 [24]. Second, it enhances precision. The results show that the 39 cases diagnosed as prostate cancer by the combination of IFSE and RMI were in complete agreement with the final diagnosis based on RARP specimens, achieving a diagnostic detection rate of 100% for prostate cancer. In contrast, the sensitivity of IFSE alone was 94.87%. Moreover, the concordance rate for GS between IFSE alone and final RARP pathology was 81.08%, whereas the concordance rate for GS achieved by combining IFSE with RMI was as high as 97.44%. Therefore, their combination improves the accuracy of intraoperative frozen section diagnosis, compensating for the sensitivity issues associated with either H&E-stained frozen sections alone or rapid IHC alone [17]. Compared to traditional IHC, this combined approach demonstrates high diagnostic consistency while significantly reducing processing time. Finally, it offers high efficiency. The mixed primary antibody incubation technique compresses the IHC protocol to within 20 min, allowing it to be completed concurrently with H&E staining of the IFSE without prolonging intraoperative waiting time. In this study, the turnaround time for obtaining a pathology report using the combined approach was 29.93 ± 2.35 min, comparable to the time previously reported for rapid frozen section examination alone [7].

Additionally, a preoperative-intraoperative multi-dimensional assessment was implemented: only high-risk suspected patients (PSA > 10 ng/mL, PI-RADS ≥ 4) were enrolled after preoperative MDT screening, and intraoperative diagnosis was confirmed only when frozen section morphology, RMI results, gross prostate observation and preoperative imaging were consistent. This comprehensive system maximized diagnostic specificity, ensuring the safety margin of surgical decision-making and the clinical rationality of our integrated diagnostic-therapeutic model.

In the integrated diagnostic- therapeutic model for prostate cancer established in this study based on the combination of RMI and IFSE, 39 patients were diagnosed with prostate cancer, and all were subsequently confirmed to have prostate cancer by final pathology of the RARP specimens. However, among the 11 patients with negative combined pathology from the targeted biopsy session in this study, 4 cases were missed diagnoses, all identified by subsequent systematic biopsy. Notably, conventional IHC performed on the remaining frozen tissue from these 4 cases was also negative, indicating that the missed diagnoses were attributable to the biopsy technique and sampling strategy itself, rather than a failure of the RMI technology, which remained accurate and reliable. Among the 50 patients enrolled, excluding the 4 missed by targeted biopsy, all required only a single hospital admission. The average hospital stay was 7.31 ± 1.41 days, significantly shorter than the duration reported in previous studies employing the traditional prostate cancer management model [8]. More importantly, this novel integrated model spares patients the psychological burden associated with waiting for conventional post-biopsy pathology results or awaiting RP surgery, reflecting a patient-centered, humane approach to care.

This study has several limitations that warrant acknowledgment. First, as a single-center prospective study with a modest sample size, the external validity and generalizability of our findings may be limited, and confirmation via large-scale, multicenter investigations is therefore needed. Second, the follow-up duration of the current study is relatively short, precluding an assessment of the long-term efficacy, safety profile and tumor recurrence risk of this integrated diagnostic-therapeutic model, as well as long-term changes in patients’ psychological status and quality of life; these outcomes require further verification with extended follow-up data. Third, no parallel control group receiving the conventional diagnostic-therapeutic paradigm was included in this study. Thus, the superiorities of the integrated model in terms of long-term oncological prognosis, cost-effectiveness and perioperative complication rates cannot be definitively established and require further validation in controlled studies.

In conclusion, the application of RMI as an adjunct to IFSE in prostate biopsy tissue facilitates the establishment of a more precise integrated diagnostic- therapeutic model for prostate cancer, demonstrating considerable potential for clinical application and broader dissemination.

## Data Availability

The data can be made available on reasonable request from the authors.
